# Supplementary material for: Inhibiting adipose tissue M1 cytokine expression decreases DPP4 activity and insulin resistance in a type 2 diabetes mellitus mouse model
Source: PLoS One. 2021 May 27;16(5):e0252153. doi: 10.1371/journal.pone.0252153 (PMC8158933; doi:10.1371/journal.pone.0252153)
Supplement: S1 Table — (DOC) [file pone.0252153.s002.doc]

**Supporting information**

| **S2 Table: Primer sequences for qPCR** | | |
| --- | --- | --- |
| **Gene** | **Forward (5’ -3’)** | **Reverse (5’ -3’)** |
| *Ccl2* | CCACAACCACCTCAAGCACTTC | AAGGCATCACAGTCCGAGTCAC |
| *Dpp4* | GGGTCTTATGCCTGTGCCTT | TCAGTAGAGCGAAGGGGTCA |
| *Fmo3* | GGA AGAGTTGGT GAAGAC CG | CCC ACA TGC TTT GAG AGG AG |
| *Icam* | GAGCTCAGCACTAGCACTTTGC | GTAGCGTGGGCTTGGCAC |
| *Il1b* | TGGTGTGTGACGTTCCCATT | CAGCACGAGGCTTTTTTGTTG |
| *Il6* | AAGCCAGAGTCCTTCAGAGAGA | ACTCCTTCTGTGACTCCAGCTT |
| *Il10* | CGACTGTTGCCTCTCGTACA | AGGAGGTTCACAGCCCTTTT |
| *Inos* | CAGCTGGGCTGTACAAACCTT | CATTGGAAGTGAAGGGTTTCG |
| *Tnfa* | GAAAGGGGATTATGGCTCAGG | TCACTGTCCCAGCATCTTGTG |
