# Supplementary figures and images for: Inhibiting adipose tissue M1 cytokine expression decreases DPP4 activity and insulin resistance in a type 2 diabetes mellitus mouse model
Source: PLoS One. 2021 May 27;16(5):e0252153. doi: 10.1371/journal.pone.0252153 (PMC8158933; doi:10.1371/journal.pone.0252153)

Fig 3B

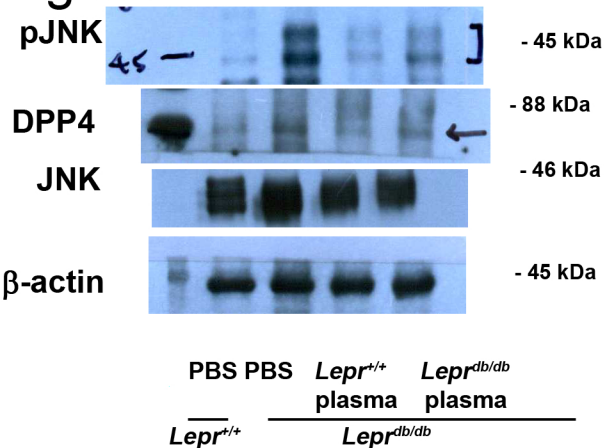

Fig 4B

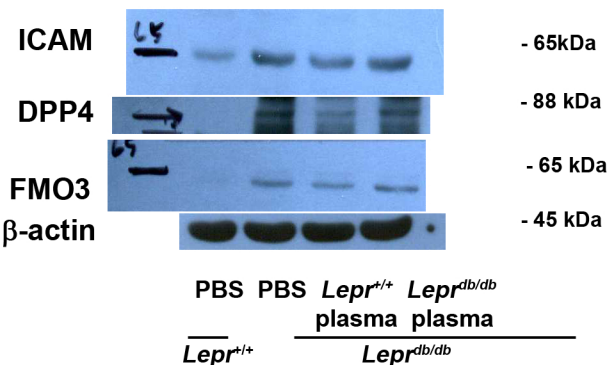

Fig 7A

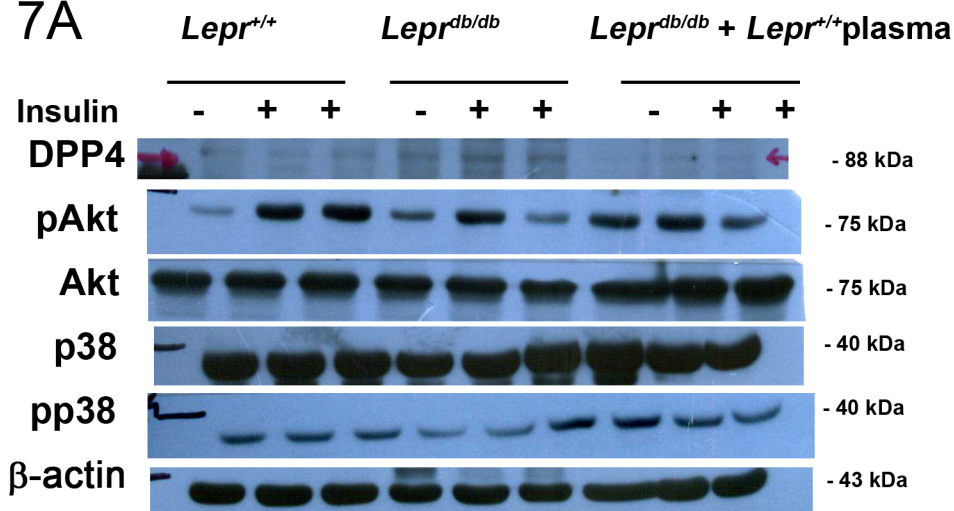

Supplement: S1 Raw images — (PDF) [file pone.0252153.s003.pdf]
